# Evaluation of injection-site-related adverse events with galcanezumab: a post hoc analysis of phase 3 studies in participants with migraine

**DOI:** 10.1186/s12883-020-01775-4

**Published:** 2020-05-19

**Authors:** Virginia L. Stauffer, Shufang Wang, Jo Bonner, ByungKun Kim, Rohit Bhandari, Kathleen A. Day, Angelo Camporeale

**Affiliations:** 1 Lilly Research Laboratories, Lilly Corporate Center, Indianapolis, IN USA; 2 St. Louis University Hospital, St Louis, USA; 3 Nowon Eulji Medical Center, Seoul, Republic of Korea; 4 Eli Lilly Services India Private Limited, Bangalore, India; 5 Eli Lilly Italia, Sesto Fiorentino, Italy

**Keywords:** Injection-site reaction, Monoclonal antibody, Migraine, Physiological, Formulation factors, Calcitonin gene-related peptide

## Abstract

**Background:**

Injection-site reactions have been reported with biologicals. In this post hoc analysis of Phase 3 studies in participants with migraine, we provide a comprehensive overview and detailed summary of injection-site reaction with galcanezumab.

**Methods:**

Data were obtained from two randomised clinical studies in participants with episodic migraine (EVOLVE-1 and EVOLVE-2), one randomised study in participants with chronic migraine (REGAIN) and one open-label study (Study CGAJ) in participants with episodic or chronic migraine. The injection-site reactions were measured for two different cohorts: 1) six-month double-blind treatment phase in the EVOLVE-1 and EVOLVE-2 studies and three-month double-blind treatment phase in the REGAIN study, where participants received placebo and galcanezumab (placebo-controlled analysis set); 2) three month double-blind (Month 0 to Month 3; 1:1:placebo:galcanezumab) + 9 months open-label extension phase (Month 3 to Month 12) of REGAIN and twelve month open-label phase of Study CGAJ, where participants received only galcanezumab (galcanezumab exposure analysis set).

**Results:**

A total of 477 participants in the placebo-controlled analysis set (galcanezumab 240 mg, 166/730 [22.7%]; galcanezumab 120 mg, 128/705 [18.2%]; placebo, 183/1451 [12.6%]) reported at least one injection-site reaction. Most of the injection-site reactions were reported as injection-site pain, unspecified injection-site reaction, injection-site erythema, and injection-site pruritus. The incidence of injection-site pain was highest among all reported injection-site reactions and were reported with similar frequency by participants receiving galcanezumab (galcanezumab 120 mg, 10.1%; galcanezumab 240 mg, 11.6%) and placebo (9.5%) and was the most common injection-site reaction reported within 60 min of injection (~ 86% of participants). The frequency of unspecified injection-site reaction, injection-site erythema and injection-site pruritus was significantly (*P* < 0.001) higher in participant receiving galcanezumab versus placebo. In the galcanezumab exposure analysis set participants received up to 12 doses and the frequency of injection-site reactions reported for both doses combined was 21.8%. The reporting of injection-site reactions did not increase with the number of doses received. No ISR-related serious adverse events were reported in both the placebo-controlled and galcanezumab exposure analysis sets.

**Conclusions:**

The most common adverse event of galcanezumab is injection-site reactions. However, these events were generally mild-to-moderate in severity, non-serious, resolved spontaneously, and discontinuations due to injection-site reactions were low (1%).

## Background

People with migraine have been shown to have elevated blood levels of calcitonin gene-related peptide (CGRP) and targeting the CGRP pathway using antibodies has been demonstrated to be effective in preventing migraine attacks [1–4]. Three such biological therapies including galcanezumab have been approved as preventive treatments for migraine [5]. Galcanezumab, erenumab and fremanezumab are administered subcutaneously, and adverse events (AEs) related to injection-sites were the most frequently reported AEs in their respective Phase 3 programs [6–8]. Injection-site reactions are local skin reactions occurring after an injection and include injection-site pain, erythema, pruritus and induration [6–8]. The aetiology of injection-site-related adverse events maybe multifocal, ranging from immunological to non-immunological factors including injection volume, temperature, pH, speed of injection, needle size, and injection excipients [9, 10].

Efficacy of galcanezumab was demonstrated in pivotal phase 3 studies in patients with episodic or chronic migraine [4, 11–13]. In the phase 3 studies, injection-site reactions were the most frequently reported AEs with galcanezumab treatment [4, 11, 12, 14].

Therefore, in this post hoc analysis of Phase 3 migraine studies of galcanezumab, we provide a comprehensive overview and detailed summary of injection-site reactions.

## Methods

Data were obtained from two randomised clinical studies in participants with episodic migraine (EVOLVE-1, clinicaltrials.gov identifier: NCT02614183, https://clinicaltrials.gov/ct2/show/NCT02614183; EVOLVE-2, clinicaltrials.gov identifier: NCT02614196, https://clinicaltrials.gov/ct2/show/NCT02614196), one randomised study in participants with chronic migraine (REGAIN, clinicaltrials.gov identifier: NCT02614261, https://clinicaltrials.gov/ct2/show/NCT02614261) and one open-label study (CGAJ, clinicaltrials.gov identifier: NCT02614287, https://clinicaltrials.gov/ct2/show/NCT02614287) in participants with episodic or chronic migraine. All clinical trials were first posted on 25 November 2015, and before enrolling the first patient. The EVOLVE and REGAIN studies were designed to study efficacy and safety of galcanezumab, and the open-label Study CGAJ was designed primarily to assess long-term safety. The study designs of all four studies have been previously published [4, 11, 12, 14].

Briefly, EVOLVE-1 and EVOLVE-2 studies had a 6-month double-blind (DB) treatment phase wherein participants were randomised 2:1:1 to receive monthly placebo, galcanezumab 120 mg or galcanezumab 240 mg. The REGAIN study had a 3-month DB treatment phase, wherein participants were randomised 2:1:1 to receive monthly placebo, galcanezumab 120 mg, or galcanezumab 240 mg. The DB phase was followed by an optional nine month open-label extension (OLE) phase wherein either galcanezumab 120 mg or galcanezumab 240 mg was administered based on the investigator’s discretion. Study CGAJ comprised a 12-month open-label (OL) phase wherein participants were randomised 1:1 to receive galcanezumab 120 mg or galcanezumab 240 mg. All four studies had a four-month safety follow-up period immediately after the treatment phase.

In the EVOLVE and REGAIN studies, study site personnel administered the injections using prefilled syringes. In Study CGAJ, injections were self-administered by participant or caregiver using prefilled syringes for up to 9 months, and then participants were switched to self-administer galcanezumab using an autoinjector [15]. Notably the autoinjector was not available for administration at the time of study initiation and was available approximately 1 year after the start of the study. As such all participants in Study CGAJ used an autoinjector from approximately Month 10 onwards. All participants and caregivers in the Study CGAJ who continued the treatment were trained to use the autoinjector before switching at Month 9 [15].

### Evaluation of injection site reactions

Two cohorts from the integrated analysis sets of four studies (EVOLVE-1, EVOLVE-2, REGAIN and CGAJ) were analysed. These studies included: 1) all participants from the six-month DB treatment phase in the EVOLVE-1 and EVOLVE-2 studies and three-month DB treatment phase in the REGAIN study (placebo-controlled analysis set); 2) galcanezumab-treated participants from the DB + OLE phase of REGAIN, Month 0 to Month 12, and OL phase of Study CGAJ, Month 0 to Month 12, where participants received galcanezumab (galcanezumab exposure analysis set). Of note, participants who were randomized to placebo in the REGAIN study received up to 9 monthly doses of galcanezumab and participants who were randomized to galcanezumab received up to 12 monthly doses of galcanezumab.

The injection-site reactions are treatment-emergent adverse events (TEAEs) that occurred or worsened at any time after treatment initiation and were coded based on the Medical Dictionary for Regulatory Activities (https://www.meddra.org/). The injection-site reactions (plural) refer to the high-level grouping of different preferred terms (PT) used to describe various injection-site reactions. The reported verbatim terms collected during the study was mapped to a PT that best characterised the reported adverse event at the injection-site, such as “pain”, “erythema”, “rash” and “swelling”. When the site recorded the event as “injection-site reaction” the event was mapped to an unspecified PT of injection-site reaction (singular). An injection-site adverse event follow-up form was used to further characterise the reported unspecified event of injection-site reaction.

### Statistical analysis

The placebo-controlled analysis set was used to evaluate injection-site reactions for up to six-month of DB treatment and the galcanezumab exposure analysis set was used to evaluate injection-site reactions up to 12 months of galcanezumab treatment.

For both analysis sets, the number of participants with injection-site reactions, discontinuations due to AEs, and serious AEs (SAEs) were summarized. For the placebo-controlled analysis set, inferential statistics was provided. Treatment comparisons were evaluated using the Cochran-Mantel-Haenszel test stratified by study. In addition, patients with injection-site reactions were summarized by maximum severity as “mild”, “moderate” or “severe”. The reaction timings from the last injection to the occurrence of injection-site reactions were summarised as follows: immediate reaction (< 60 min); acute reaction (1 to 6 h); delayed reaction (classified as injection-site reactions post 6 h on the day of the injection up to 14 days after the day of injection); and reactions ≥14 days. The duration of injection-site reactions were also summarized.

For the galcanezumab exposure data set, to determine if injection-site reactions excluding pain were reported more frequently with repeated doses of galcanezumab, the number of TEAEs were assessed by the total number of doses received.

## Results

### Demographics

All 3156 participants from the EVOLVE-1, EVOLVE-2 and REGAIN studies were included in this post hoc analysis (placebo, *n* = 1451; galcanezumab 120 mg, *n* = 840; galcanezumab 240 mg, *n* = 865). In this integrated analysis set of EVOLVE-1, EVOLVE-2 and REGAIN studies, participants were predominantly women (placebo, 1237/1451 [85.3%]; galcanezumab 120 mg, 599/705 [85.0%]; galcanezumab 240 mg, 609/730 [83.4%]), with a mean age of ~ 41 years (Table 1). On an average, participants were diagnosed as having migraine ~ 20 years prior to study enrolment. Baseline demographics of race, region and comorbid conditions were similar across treatment groups. The most common comorbid conditions (occurring in > 10% of all participants), included seasonal allergy, drug hypersensitivity, insomnia, anxiety, depression, and back pain.

**Table 1:** Baseline demographics and comorbid conditions in EVOLVE-1, EVOLVE-2 and REGAIN studies

	Placebo (N = 1451)	GMB 120 mg (*N* = 705)	GMB 240 mg (*N* = 730)
**Demographics**			
Age, mean years, (SD)	41.8 (11.6)	40.8 (11.5)	40.4 (11.9)
Gender (female), n (%)	1237 (85.3)	599 (85.0)	609 (83.4)
Race, n (%)			
White	1112 (76.6)	545 (77.3)	^a^555 (76.1)
Black or African American	117 (8.1)	53 (7.5)	^a^59 (8.1)
Asian	89 (6.1)	48 (6.8)	^a^42 (5.8)
American Indian or native Alaska	24 (1.7)	10 (1.4)	^a^16 (2.2)
Native Hawaiian or other pacific islander	2 (0.1)	0	^a^4 (0.6)
Multiple	107 (7.4)	49 (7.0)	^a^53 (7.3)
Region, n (%)			
North America	977 (67.3)	472 (67.0)	496 (68.0)
Europe	262 (18.1)	126 (17.9)	131 (18.0)
Other	212 (14.6)	107 (15.2)	103 (14.1)
Years since migraine diagnosis, mean (SD)	21.1 (12.7)	20.7 (12.5)	19.6 (12.3)
**Comorbid conditions that occurred in > 10% of all participants, n (%)**			
Seasonal allergy	307 (21.2)	158 (22.4)	122 (16.7)
Drug hypersensitivity	247 (17.0)	123 (17.5)	128 (17.5)
Insomnia	165 (11.4)	89 (12.6)	78 (10.7)
Anxiety	166 (11.4)	82 (11.6)	81 (11.1)
Depression	181 (12.5)	84 (11.9)	79 (10.8)
Back pain	151 (10.4)	62 (8.8)	75 (10.3)

**Abbreviations:***GMB* galcanezumab, *ISR* injection-site reaction, *N* number of participants in the intent-to-treat population, *n* number of participants within each specific category, *SD* standard deviation

^a^N = 729

Note: All values are for injection-site reactions during the double-blind treatment phase from study start up to 6 months for EVOLVE-1 and EVOLVE-2 and up to 3 months for REGAIN

In Study CGAJ, participants were predominantly women (galcanezumab 120 mg, 110/135 [81.5%]; galcanezumab 240 mg 113/135 [83.7%]), with a mean age of ~ 42 years (mean [SD]: galcanezumab 120 mg, 40.2 [11.7]; galcanezumab 240 mg, 43.7 [11.0]) and a diagnosis of migraine of ~ 20 years prior to study enrolment (mean [SD]: galcanezumab 120 mg, 20.2 [12.4]; galcanezumab 240 mg, 21.3 [12.5]). The detailed demographics are published elsewhere [14].

### Injection-site reactions during double-blind phase of EVOLVE-1, EVOLVE-2 and REGAIN studies

During the DB treatment phase of EVOLVE-1, EVOLVE-2 and REGAIN studies, 477 (477/2886, 16.5%) participants reported at least one injection-site reaction (galcanezumab 240 mg, 166/730 [22.7%]; galcanezumab 120 mg, 128/705 [18.2%]; placebo, 183/1451 [12.6%]). The injection-site reactions were significantly higher (*P* ≤ 0.001) with galcanezumab 240 mg or 120 mg compared with placebo (Table 2). The most commonly (≥2%) observed injection-site reactions were injection-site pain, unspecified injection site reaction, injection-site erythema, and injection-site pruritus. These were reported by higher proportion of participants in galcanezumab 240 mg and 120 mg groups compared with placebo (Table 2).

**Table 2:** Summary of injection-site reactions in pooled EVOLVE-1, EVOLVE-2, and REGAIN (placebo-controlled analysis set)

	Placebo (*N* = 1451)	GMB 120 mg (*N* = 705)	GMB 240 mg (*N* = 730)
Any/all ISRs, n (%)	183 (12.6)	128 (18.2)	166 (22.7)
ISRs excluding pain, n (%)	60 (4.1)	70 (9.9)	106 (14.5)
ISRs, n (%)			
IS pain	138 (9.5)	71 (10.1)	85 (11.6)
Unspecified ISR	14 (1.0)	22 (3.1)	45 (6.2)
IS erythema	20 (1.4)	20 (2.8)	29 (4.0)
IS pruritus	2 (0.1)	15 (2.1)	24 (3.3)
IS bruising	9 (0.6)	4 (0.6)	10 (1.4)
IS swelling	1 (0.1)	8 (1.1)	4 (0.6)
IS rash	2 (0.1)	6 (0.9)	4 (0.6)
IS induration	1 (0.1)	3 (0.4)	3 (0.4)
IS discomfort	3 (0.2)	3 (0.4)	2 (0.3)
IS hematoma	7 (0.5)	1 (0.1)	3 (0.4)
IS hypersensitivity	0	1 (0.1)	3 (0.4)
IS mass	0	3 (0.4)	0
IS haemorrhage	2 (0.1)	1 (0.1)	1 (0.1)
IS inflammation	0	1 (0.1)	1 (0.1)
IS irritation	3 (0.2)	1 (0.1)	1 (0.1)
IS urticarial	1 (0.1)	1 (0.1)	1 (0.1)
IS discoloration	0	0	1 (0.1)
IS oedema	1 (0.1)	1 (0.1)	0
IS papules	0	1 (0.1)	0
IS vesicles	0	0	1 (0.1)
IS warmth	1 (0.1)	0	0

**Abbreviations:***GMB* galcanezumab, *n* number of participants within each specific category, *IS* injection-site, *ISR* injection-site reaction, *N* number of participants in the intent-to-treat population

Note: All values are for injection-site reactions during the double-blind treatment phase from study start up to 6 months for EVOLVE-1 and EVOLVE-2 and up to 3 months for REGAIN

Of the 67 galcanezumab-treated participants who reported an unspecified injection site reaction (galcanezumab 120 mg, 22/705 [3.1%]; galcanezumab 240 mg, 45/730 [6.2%]), all participants completed at least one follow-up form which was used to further characterise the reported unspecified injection site reaction. Of these 67 participants, 59.1% (13/22) participants on galcanezumab 120 mg reported itching, rash or redness, and injection-site hardening. Itching, rash or redness, and injection-site hardening were reported by 60.0% (27/45), 84.4% (38/45), and 44.4% (20/45) participants on galcanezumab 240 mg, respectively.

Among patients who reported injection-site reactions, most reported injection-site reactions of mild-to-moderate severity (Table 3). No injection-site reactions were reported as SAEs. Overall seven participants discontinued due to injection-site reactions (galcanezumab 120 mg, *n* = 2; galcanezumab 240 mg, *n* = 5). Among these participants, four participants discontinued due to moderate unspecified injection-site reaction (galcanezumab 120 mg, 1/705 [0.1%]; galcanezumab 240 mg, 3/730 [0.4%]). The remaining three patients discontinued due to moderate injection-site pain (galcanezumab 240 mg, 1/1451 [0.1%]), severe injection-site erythema (galcanezumab 120 mg, 1/705 [0.1%]), and moderate injection-site swelling (galcanezumab 240 mg, 1/730 [0.1%]) Table 3.

**Table 3:** Summary of adverse events related to injection-site reaction (≥2%) in pooled EVOLVE-1, EVOLVE-2 and REGAIN (placebo-controlled analysis set)

	IS pain			Unspecified ISR			IS erythema			IS pruritus		
	Placebo (*N* = 1451)	GMB 120 mg (*N* = 705)	GMB 240 mg (*N* = 730)	Placebo (*N* = 1451)	GMB 120 mg (*N* = 705)	GMB 240 mg (*N* = 730)	Placebo (*N* = 1451)	GMB 120 mg (*N* = 705)	GMB 240 mg (*N* = 730)	Placebo (*N* = 1451)	GMB 120 mg (*N* = 705)	GMB 240 mg (*N* = 730)
Participants with ≥2% TEAE, n (%)	138 (9.5)	71 (10.1)	85 (11.6)	14 (1.0)	22 (3.1)	45 (6.2)	20 (1.4)	20 (2.8)	29 (4.0)	2 (0.1)	15 (2.1)	24 (3.3)
Maximum severity of ISRs n (%)												
Mild	79 (5.4)	41 (5.8)	50 (6.9)	8 (0.6)	8 (1.1)	23 (3.2)	11 (0.8)	13 (1.8)	21 (2.9)	2 (0.14)	10 (1.4)	12 (1.6)
Moderate	41 (2.8)	21 (3.0)	29 (4.0)	6 (0.4)	14 (2.0)	20 (2.7)	9 (0.6)	5 (0.7)	5 (0.7)	0	2 (0.3)	6 (0.8)
Severe	18 (1.2)	9 (1.3)	6 (0.8)	0	0	2 (0.3)	0	2 (0.3)	3 (0.4)	0	3 (0.4)	6 (0.8)
SAE of ISR, n (%)	0	0	0	0	0	0	0	0	0	0		0
^a^Discontinued due to ISR, n (%)	0	0	1 (0.1)	0	1 (0.1)	3 (0.4)	0	1 (0.1)	0	0	0	0
Median duration of ISR event, days (SD)	1.6 (8.4)	1.2 (1.0)	1.5 (2.7)	1.2 (0.8)	5.3 (9.3)	3.7 (3.9)	3.8 (16.0)	6.5 (9.4)	4.6 (5.6)	1.0 (0.00)	6.1 (7.5)	6.2 (7.2)

**Abbreviations:***AE* adverse events, *GMB* galcanezumab, *IS* injection-site, *ISR* injection-site reaction, *N* number of participants in the safety population, *n* number of participants within each specific category, *SAE* serious adverse event, *SD* standard deviation, *TEAE* treatment-emergent adverse event

^a^one galcanezumab 240 mg participant discontinued due to injection-site swelling

Note: All values include AEs related to ISR during the double-blind treatment phase from study start up to 6 months for EVOLVE-1, and EVOLVE-2 and up to 3 months for REGAIN

Injection-site pain was the most common immediate injection-site reaction reported within 60 min of injection) and was observed in approximately 86% of participants reporting injection-site pain (Table 4). Majority of unspecified-injection-site reaction (placebo, 100.0%; galcanezumab, 88.0%), injection-site erythema (placebo, 95.0%; galcanezumab, 79.0%) and injection-site pruritus (placebo, 100%; galcanezumab. 74.4%) occurred on the day of injection (Table 4). Only two participants on galcanezumab (galcanezumab 120 mg, 1 [1.4%] and galcanezumab 240 mg, 1 [1.2%]) had a reaction after 14 days. Majority of the injection-site reactions occurred on the day of injection and were resolved, either on the same day or a few days afterwards (mean [SD] duration in days, injection-site pain: placebo, 1.6 [8.35]; galcanezumab 120 mg, 1.2 [1.01]; galcanezumab 240 mg, 1.5 [2.68] and injection-site reaction excluding pain: placebo, 2.0 [8.76]; galcanezumab 120 mg, 2.8 [5.46]; galcanezumab 240 mg, 2.7 [4.17]).

**Table 4:** Time to onset of injection-site reactions in pooled EVOLVE-1, EVOLVE-2 and REGAIN (placebo-controlled analysis set)

		n	All	Immediate response (< 60 min)	Acute reaction (1 to 6 h)	Delayed reaction (6 to 14 h)		Reaction > 14 days	Total on day of injection	Total after day of injection
						On day of injection	After day of injection			
IS pain, n (%)	Placebo	1451	134 (9.2)	130 (97.0)	0 (0.0)	3 (2.2)	1 (0.7)	0 (0.0)	133 (99.3)	1 (0.7)
	GMB 120 mg	705	70 (9.9)	62 (88.6)	3 (4.3)	1 (1.4)	3 (4.3)	1 (1.4)	66 (94.3)	4 (5.7)
	GMB 240 mg	730	84 (11.5)	71 (84.5)	3 (3.6)	6 (7.1)	3 (3.6)	1 (1.2)	80 (95.2)	4 (4.8)
Unspecified ISR, n (%)	Placebo	1451	14 (1.0)	13 (92.9)	1 (7.1)	0 (0.0)	0 (0.0)	0 (0.0)	14 (100.0)	0 (0.0)
	GMB 120 mg	705	22 (3.1)	8 (36.4)	5 (22.7)	5 (22.7)	4 (18.2)	0 (0.0)	18 (81.8)	4 (18.2)
	GMB 240 mg	730	44 (6.0)	13 (29.5)	14 (31.8)	13 (29.5)	4 (9.1)	0 (0.0)	40 (90.9)	4 (9.1)
IS erythema, n (%)	Placebo	1451	20 (1.4)	18 (90.0)	1 (5.0)	0 (0.0)	1 (5.0)	0 (0.0)	19 (95.0)	1 (5.0)
	GMB 120 mg	705	20 (2.8)	7 (35.0)	5 (25.0)	4 (20.0)	4 (20.0)	0 (0.0)	16 (80.0)	4 (20.0)
	GMB 240 mg	730	28 (3.8)	7 (25.0)	11 (39.3)	4 (14.3)	6 (21.4)	0 (0.0)	22 (78.6)	6 (21.4)
IS pruritus, n (%)	Placebo	1451	2 (0.1)	2 (100.0)	0 (0.0)	0 (0.0)	0 (0.0)	0 (0.0)	2 (100.0)	0 (0.0)
	GMB 120 mg	705	15 (2.1)	3 (20.0)	3 (20.0)	6 (40.0)	3 (20.0)	0 (0.0)	12 (80.0)	3 (20.0)
	GMB 240 mg	730	24 (3.3)	2 (8.3)	10 (41.7)	5 (20.8)	7 (29.2)	0 (0.0)	17 (70.8)	7 (29.2)

**Abbreviations:***GMB* galcanezumab, *IS* injection-site, *ISR* injection-site reaction, *n* number of patients with non-missing reaction timings

Note: All values are for ISR during the double-blind treatment phase from study start up to 6 months for EVOLVE-1 and EVOLVE-2 and up to 3 months for REGAIN

### Injection-site reactions during long-term treatment with galcanezumab in REGAIN and study CGAJ

During the long-term exposure of galcanezumab, 289 (289/1326, 21.8%) participants reported AEs related to injection-sites. The most frequently reported injection-site reactions were injection-site pain (galcanezumab pooled, 108/1326 [8.1%]), unspecified injection-site reaction (galcanezumab pooled, 103/1326 [7.8%]), injection-site erythema (galcanezumab pooled, 62/1326 [4.7%]), and injection-site pruritus (galcanezumab pooled, 30/1326 [2.3%]; Table 5). Overall nine patients discontinued the treatment due to injection-site reactions (Study CGAJ, *n* = 5; REGAIN, *n* = 4). All discontinuations were observed following multiple doses of galcanezumab (fourth, *n* = 1; fifth injection, n = 1; sixth injection, n = 1; seventh injection, *n* = 3; ninth injection, n = 1; 10th injection, *n* = 2).

**Table 5:** Injection-site reactions by monthly dosing interval among pooled galcanezumab-treated participants in CGAJ and REGAIN studies, ≥1% incidence (galcanezumab exposure analysis set)

Participants with ≥ 1 ISRs	Total, n (%)
**N**	**1326**
IS pain	**108 (8.1)**
Unspecified ISRs	**103 (7.8)**
IS erythema	**62 (4.7)**
IS pruritus	**30 (2.3)**
IS bruising	**25 (1.9)**
IS rash	**18 (1.4)**
IS hematoma	**14 (1.1)**

**Abbreviations:***IS* injection-site, *ISR* injection-site reaction,  *N* number of galcanezumab-treated participants, *n* number of participants within each specific category

Note: All values are for ISRs from study start up to 12 months for REGAIN and CGAJ studies; pooled galcanezumab = galcanezumab 120 mg + galcanezumab 240 mg

To evaluate if galcanezumab-treated patients reported multiple injection-site reactions (excluding pain) over consecutive monthly injections, the number of TEAEs related to injection-sites (excluding pain) by total number of doses is provided in Table 6. In summary 81% of patients received 9 doses or more of galcanezumab and most patients reported 1 to 3 events with monthly injections over 9 to 12 months suggesting that the reporting of injection-site reactions (excluding pain) did not increase with multiple dose administrations.

**Table 6:** Summary of participants with single or multiple injection-site reactions excluding pain among pooled galcanezumab-treated participants in Study CGAJ and REGAIN (galcanezumab exposure analysis set)

Injection-site reactions, n (%)			
Total doses received (***N*** = 1326)	1 to 3	4 to 9	> 10
1 (26)	2 (7.7)	0	0
2 (36)	2 (5.6)	0	0
3 (32)	1 (3.1)	1 (3.1)	0
4 (35)	6 (17.1)	0	0
5 (32)	6 (18.8)	0	0
6 (39)	5 (12.8)	3 (7.7)	0
7 (29)	7 (24.1)	2 (6.9)	0
8 (26)	7 (26.9)	0	0
9^a^ (433)	30 (6.9)	12 (2.8)	2 (0.5)
10 (9)	3 (33.3)	1 (11.1)	0
11 (11)	2 (18.2)	0	0
12 (618)	97 (15.7)	34 (5.5)	6 (1.0)

^a^the number of patients receiving up to nine doses of galcanezumab, includes patients from both Study CGAJ (Month 0 to Month 12) and REGAIN (galcanezumab, Month 0 to Month 12; and placebo patients who had received placebo treatment from Month 0 to Month 3 and then initiated galcanezumab in the open-label extension phase and could receive up to nine doses of galcanezumab

**Abbreviations:***N* number of galcanezumab-treated participants with a specific number of galcanezumab doses, *n* number of patients within each specific category

## Discussion

In Phase 3 studies demonstrating efficacy and safety of galcanezumab as a treatment option for management of migraine, injection-site reactions were the most commonly reported AEs. The incidence of injection-site pain was highest among all reported injection-site reactions and was reported equally by participants receiving galcanezumab and placebo. Unspecified injection-site reaction, injection-site erythema and injection-site pruritus were significantly (*P* < 0.001) higher in participants being treated with galcanezumab compared with placebo. Overall, the incidence of injection-site reactions were higher in the galcanezumab dose groups (240 mg > 120 mg) compared with placebo-treated participants and appear to be driven by AEs related to injection-sites including unspecified injection-site reaction, injection-site erythema, and injection-site pruritus. These reactions are commonly observed with other approved monoclonal antibodies for subcutaneous use, including adalimumab, denosumab, ixekizumab, and canakinumab [16–18] as well as CGRP monoclonal antibodies [7, 19, 20].

In this post hoc analysis of four Phase 3 studies, we provide a more comprehensive summary of injection-site reactions with short-term and long-term exposure to galcanezumab. Of the most commonly reported injection-site reactions in galcanezumab-treated participants, 70 to 100% were reported on the day of injection, were generally mild-to-moderate in severity, non-serious, and resolved spontaneously. In total 16/1705 (< 1%) treatment discontinuations were observed due to injection-site reactions; seven were observed in placebo-controlled analysis set and nine (Study CGAJ, *n* = 5; REGAIN, *n* = 4) were observed in the galcanezumab exposure analysis set. Most participants reported onset of injection-site pain within 60 min of injection, and approximately 50% of injection-site pain was reported as injection-site burning. Of interest, 17.5% of patients reported a history of hypersensitivity, however only 5.2% of those patients reported injection-site related AEs other than pain. During exposure to galcanezumab up to 12 months of treatment, the number of injection-site reactions reported by the same patient does not increase with more doses received.

Notably, therapeutic monoclonal antibodies (mAb), including galcanezumab, have the potential to have anti-drug antibody (ADA) formation, which in turn can block the active mAb site. The formation of ADA can be associated with changes to pharmacokinetic and pharmacodynamic parameters, resulting in a range of effects from no clinically important effects to reduced drug efficacy and/or increased risk of adverse events (AEs) [21, 22]. A recently published post hoc analysis of EVOLVE-1, EVOLVE-2, REGAIN and CGAJ studies, showed that approximately 2.6 to 12.4% of galcanezumab-treated patients developed treatment-emergent ADA. The characteristics of the immune response observed were not related to any clinically meaningful consequences on pharmacokinetic, pharmacodynamics, efficacy, or safety of galcanezumab. There were no differences in injection-site reactions reported in patients who developed treatment emergent ADA compared to those who did not.

Therapeutic proteins are typically administered in the subcutaneous tissue, especially when the treatment is required frequently, on a long-term basis and requires self-administration [23]. Injection-site reactions with therapeutic proteins may arise from participant-related factors such as variability in injection speed (association of injection-site pain with fast injections), participant-to-participant differences in pain tolerance. The injection-site reactions with therapeutic proteins may also arise from variations in formulation-related factors such as formulation temperature (which should ideally be close to body temperature), type of injectable device used (prefilled syringe versus autoinjector), injection volume (ideally should be ≤3 mL [24]), pH (ideally should be physiological) and excipients [23, 25]. For galcanezumab, the pH of the formulation is between 5.3 and 6.3, the injection volume is 1 mL, the inactive excipient contains polysorbate 80, L-histidine, histidine hydrochloride monohydrate, and sodium chloride [26]. During short-term exposure to galcanezumab, we observed similar incidence of injection-site pain between placebo- and galcanezumab-treated participants. This may explain the possibility that formulation-based factors, such as non-physiological pH (i.e. < 7) and presence of polysorbate 80 could have resulted in injection-site pain. These findings are in agreement with the earlier findings of Kaiser et al. (2012), who have cited injection-site pain associated with Kineret (anakinra) owing to its non-physiological pH and presence of polysorbate 80 in the formulation [25, 27].

This post hoc analysis, which was not pre-specified at the time of design of the EVOLVE-1, EVOLVE-2, REGAIN and Study CGAJ, limits its ability to make definitive conclusions. There could also be some under-reporting possibly as all AEs were reported spontaneously by the study participants, and use of analgesic, and histamine creams, and potential comfort measures were not excluded. Lastly, galcanezumab injections were administered by study site personnel in the galcanezumab Phase 3 studies, with the exception of Study CGAJ. In Study CGAJ, patients self-administered galcanezumab either with the prefilled syringe or the autoinjector devices. The injection experience and tolerability of self-administration with both devices has been published elsewhere [15].

## Conclusions

In conclusion, galcanezumab-treated participants reported a significantly higher frequency of injection-site reactions versus placebo. Most of these events were self-limiting; no SAEs related to injection-site were reported in any of the Phase 3 migraine studies. Future post-marketing study evidence is warranted to augment these findings.

## Supplementary information

**Additional file 1.** List of ethics committee.

## Data Availability

Lilly provides access to all individual participant data collected during the trial, after anonymization, with the exception of pharmacokinetic or genetic data. Data are available to request 6 months after the indication studied has been approved in the US and EU and after primary publication acceptance, whichever is later. No expiration date of data requests is currently set once data are made available. Access is provided after a proposal has been approved by an independent review committee identified for this purpose and after receipt of a signed data sharing agreement. Data and documents, including the study protocol, statistical analysis plan, clinical study report, blank or annotated case report forms, will be provided in a secure data sharing environment. For details on submitting a request, see the instructions provided at www.vivli.org.

## Abbreviations

ADA: Anti-drug antibody
AE: Adverse events
CGRP: Calcitonin Gene-Related Peptide
DB: Double-blind
HLT: High-level term
OL: Open-label
OLE: Open-label extension
PT: Preferred term
SAEs: Serious adverse events
SD: Standard deviation
