# Supplementary material for: Evaluation of injection-site-related adverse events with galcanezumab: a post hoc analysis of phase 3 studies in participants with migraine
Source: BMC Neurol. 2020 May 19;20:194. doi: 10.1186/s12883-020-01775-4 (PMC7236916; doi:10.1186/s12883-020-01775-4)
Supplement: Supplementary file 1 — Additional file 1. List of ethics committee. [file 12883_2020_1775_MOESM1_ESM.docx]

# Additional file 1:

# List of ethics committee

The ethical review boards included Quorum Review Inc., IRB Services, Montreal Neurological Institute and Hospital, Cedars Sinai Medical Center, North West – Liverpool East – Research Ethics Committee, Isala Klinieken METC, Hospital Universitari Vall d’Hebron - Comité Ético de Investigación Clínica, Dean Foundation for Health Research and Education, Eticka komise IKEM a Thomayerovy nemocnice, Ethikkommission der Landesärztekammer Hessen, Comite de Etica Independiente en Invest. Clinica Dr. C Barclay, Comite de Etica del Centro de Osteopatias Medicas, Hillel Yaffe Medical Center, Rambam Medical Center, Chaim Sheba Medical, Maccabi Healthcare Services Kfar Saba ERB at Assuta Medical Center, Hallym University Dongtan Sacred Heart Hospital, Samsung Medical Center, Eulji General Hospital, Seoul National University Hospital, Korea University Guro Hospital, Kangbuk Samsung Hosp, Hallym Univ. of Medicine, Kangnam Sacred Heart Hospital, Chi-Mei Medical Center - Yung Kang, Kaohsiung Medical University Chung-Ho Memorial Hospital, Sin-Lau Hospital, Taipei Veterans General Hospital, Research Ethics Review Committee, Grupo Médico Camino S.C., Medical Care and Research, S.A. de C.V., Hospital Angeles de Culiacan, Grupo Medico Carracci, Estimulación Magnetica Transcraneal de Mexico, Baylor, Scott, & White, West Midlands – Edgbaston REC, Comitato Etico Irccs San Raffaele Pisana, Comitato Etico Interaziendale Bologna-IMOLA, Comitato Etico Area Vasta Centro Presso AOU, Comitato Etico della Provincia di Modena, Comitato Etico Ospedale San Raffaele, Eticka komise Clintrial, s.r.o., Eticka komise FN u sv. Anny v Brne, Sanatorio Allende-Cordoba, Instituto Reumatologico Strusberg, Tel Aviv Sourasky Medical Center, Thomas Jefferson University, Crescent City institutional Review Board, Office of Research Compliance - University of South Carolina, Far Eastern Memorial Hospital Research Ethics Review Committee, Commissie Medische Ethiek Universitair Ziekenhuis Brussel, Conjoint Medical Ethics Committee, CPP Sud Mediterannée V, and Egeszsegugyi Tudomanyos Tanacs.
